# Influence of Peanut Consumption on the Gut Microbiome: A Randomized Clinical Trial

**DOI:** 10.3390/nu16193313

**Published:** 2024-09-30

**Authors:** Sang Minh Nguyen, Thi Du Chi Tran, Thi Mo Tran, Cong Wang, Jie Wu, Qiuyin Cai, Fei Ye, Xiao-Ou Shu

**Affiliations:** 1 Division of Epidemiology, Department of Medicine, Vanderbilt Epidemiology Center, Vanderbilt-Ingram Cancer Center, Vanderbilt University School of Medicine, Nashville, TN 37203, USA; sang.minh.nguyen@vumc.org (S.M.N.); cong.wang@vanderbilt.edu (C.W.); jie.wu@vumc.org (J.W.); qiuyin.cai@vumc.org (Q.C.); fei.ye@vumc.org (F.Y.); 2 Vietnam Colorectal Cancer and Polyps Research, Vinmec Healthcare System, Hanoi 10000, Vietnam; v.chittd@vinmec.com (T.D.C.T.); mo.tt@vinuni.edu.vn (T.M.T.); 3 College of Health Sciences, VinUniversity (VinUni), Hanoi 67000, Vietnam; 4 Vinmec-VinUni Institute of Immunology, Hanoi 10000, Vietnam; 5 Department of Biostatistics, Vanderbilt University School of Medicine, Nashville, TN 37203, USA

**Keywords:** peanut consumption, randomized clinical trial, gut microbiome, shotgun metagenomic sequencing, Vietnam

## Abstract

**Background:** Peanut consumption could impact cardiometabolic health through gut microbiota, a hypothesis that remains to be investigated. A randomized clinical trial in Vietnam evaluated whether peanut consumption alters gut microbiome communities. **Methods**: One hundred individuals were included and randomly assigned to the peanut intervention and control groups. A total of 51 participants were provided with and asked to consume 50 g of peanuts daily, while 49 controls maintained their usual dietary intake for 16 weeks. Stool samples were collected before and on the last day of the trial. After excluding 22 non-compliant participants and those who received antibiotic treatment, 35 participants from the intervention and 43 from the control were included in the analysis. Gut microbiota composition was measured by shotgun metagenomic sequencing. Associations of changes in gut microbial diversity with peanut intervention were evaluated via linear regression analysis. Linear mixed-effects models were used to analyze associations of composition, sub-community structure, and microbial metabolic pathways with peanut intervention. We also performed beta regression analysis to examine the impact of peanut intervention on the overall and individual stability of microbial taxa and metabolic pathways. All associations with false discovery rate (FDR)-corrected *p*-values of <0.1 were considered statistically significant. **Results:** No significant changes were found in α- and β-diversities and overall gut microbial stability after peanut intervention. However, the peanut intervention led to lower enrichment of five phyla, five classes, two orders, twenty-four metabolic pathways, and six species-level sub-communities, with a dominant representation of *Bifidobacterium pseudocatenulatum*, *Escherichia coli* D, *Holdemanella biformis*, *Ruminococcus* D *bicirculans*, *Roseburia inulinivorans*, and MGYG-HGUT-00200 (*p* < 0.05 and FDR < 0.1). The peanut intervention led to the short-term stability of several species, such as *Faecalibacterium prausnitzii* F and H, and a metabolic pathway involved in nitrate reduction V (*p* < 0.05; FDR < 0.1), known for their potential roles in human health, especially cardiovascular health. **Conclusions:** In summary, a 16-week peanut intervention led to significant changes in gut microbial composition, species-level sub-communities, and the short-term stability of several bacteria, but not overall gut microbial diversity and stability. Further research with a larger sample size and a longer intervention period is needed to confirm these findings and investigate the direct impact of gut-microbiome-mediated health effects of peanut consumption. Trial registration: The International Traditional Medicine Clinical Trial Registry (ITMCTR). Registration number: ITMCTR2024000050. Retrospectively Registered 24 April 2024.

## 1. Introduction

Associations between peanut consumption and cardiovascular health have been extensively investigated in epidemiological studies and clinical trials [1,2,3]. The intake of this groundnut, although classified as a legume, was shown to improve lipid profiles, enhance glycemic control, inhibit inflammation, and lower blood pressure levels [4,5,6], which might contribute to a reduced risk of chronic diseases, including obesity, cardiovascular disease, and type 2 diabetes mellitus [7,8]. Additionally, peanut consumption was associated with decreased mortality from ischemic and hemorrhagic stroke, ischemic heart disease, and cardiovascular disease in prospective studies [9,10]. However, the biological mechanisms underlying these associations are not fully understood.

Peanuts, the most consumed groundnuts, are known as functional food owing to their potential benefits to human health; they are rich in nutrients such as unsaturated fatty acids, particularly monounsaturated fatty acids, as well as polyunsaturated fatty acids (e.g., linoleic acids), and phytochemicals, including fiber, vitamins (e.g., Vitamin E), phenolic antioxidants (e.g., resveratrol), arginine, and other bioactive compounds [11]. The mechanism through which peanut consumption confers these benefits may be related to the physiological effects of nutrients and chemical compounds, or to the role of gut microbiota [12,13]. The interaction between peanuts, their nutrients, and gut microbiota may be key moderators of human metabolism [14,15].

Consuming peanuts can impact cardiometabolic health through their influence on the gut microbiota [16]. The gut microbiota is known to play a vital role in human health. It helps maintain barrier homeostasis, protects against pathogen overgrowth, matures the immune response, influences epithelial hyperproliferation, and supports vascularization in the gastrointestinal tract [17,18,19,20,21]. Additionally, the gut microbiota regulates intestinal endocrine functions, providing a key source of energy biogenesis, biosynthesizing vitamins, modulating neurological signaling and neurotransmitters, metabolizing bile acids, reacting to or modifying specific drugs, and eliminating exogenous toxins [22,23,24,25,26]. Peanut consumption may modulate gut microbiome communities by fostering the growth of beneficial gut bacteria, such as those producing short-chain fatty acids (SCFAs), which are beneficial to human health [16]. Two recent systematic reviews reported that the intake of nuts, particularly walnuts, had a potential impact on gut microbiota diversity and was associated with several shifts in bacterial composition, such as genera capable of producing SCFAs, including *Clostridium*, *Roseburia*, *Lachnospira*, and *Dialister* [27,28]. However, these findings were inconsistent across studies [27,28]. Moreover, most research has focused on walnuts, hazelnuts, and almonds, whose nutritional compositions differ from those of peanuts [27]. Few intervention studies have investigated the influence of peanut consumption on gut microbiota [29,30].

The influence of peanut consumption on gut microbiota has yet to be investigated thoroughly. To fill this research gap, we conducted a randomized clinical trial in Vietnam, the Vietnam Peanut Trial Study, to evaluate whether peanut consumption alters gut microbiome communities.

## 2. Materials and Methods

### 2.1. Study Population

**Figure 1:**
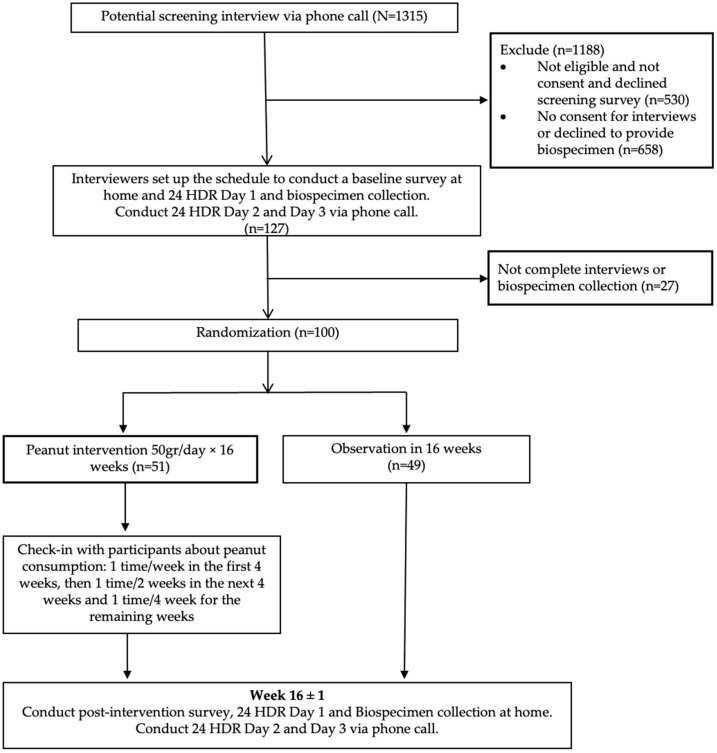
Participant flowchart: screening, randomization, and follow-up.

The Vietnam Peanut Trial Study recruited one hundred study participants and randomly assigned them to a peanut intervention group or a control group from April 2019 to Jun 2020 (The International Traditional Medicine Clinical Trial Registry: ITMCTR2024000050). The participants for the trial were recruited from a colorectal cancer screening project in Hanoi, Vietnam, known as the Vietnam Colorectal Cancer and Polyps Research (VinCAPR). The design and methods for the VinCAPR have been described in detail elsewhere [31]. Briefly, the VinCAPR project involved 103,542 residents in Hanoi aged 40 or older and used a two-step modality with an immunochemical fecal occult blood test (iFOBT) followed by a colonoscopy for those who tested positive. The potential participants were selected from the pool of screeners who tested negative for iFOBT. The inclusion criteria for the trial were as follows: (1) aged between 40 and 70; (2) did not consume peanuts or other tree nuts/seeds more than one time per week; (3) self-reported diabetes/prediabetes, but not on diabetes medications; and (4) self-reported dyslipidemia, but not on lipid- or triglyceride-lowering medications. Individuals who met any of the following conditions were not eligible for the study: (1) known or suspected allergy to peanut/peanut-containing products; (2) diagnosed with any kidney disease, liver disease, heart attack, stroke, other cardiovascular disease, or cancer; (3) drank more than two units of alcohol (two cups of wine, two shots of liquid, or two cans of beer) daily; (4) current or recent use of diabetes medications or lipid- or triglyceride-lowering medications in the past three months; (5) hospitalized in the past three months; (6) received chemotherapy, immune suppressive treatment, or antibiotics during the past three months; or (7) gained or lost more than 2.5 kg of weight in the past three months. A phone screening interview was then conducted to identify approximately 1315 potential candidates. Finally, one hundred participants were selected from the potential candidates (Figure 1). The sample size of 100 participants was determined to ensure the trial was adequately powered to detect a minimal mean difference of 0.566 standard deviations between the peanut intervention and control groups.

These one-hundred participants were randomly assigned to two arms. Fifty-one participants were provided with and asked to consume 50 g of peanuts daily for 16 weeks, while 49 controls maintained their usual dietary intake for 16 weeks. Domestic-produced plain peanuts (unsalted and unsweetened) in 50 g bags were purchased and analyzed for regulated mycotoxins in raw materials using UHPLC-MS/MS before being dispensed to the peanut intervention group [32]. All Aflatoxin B1, Aflatoxin B2, Aflatoxin G1, and Aflatoxin B2 levels were less than 0.500 ng/g in the testing samples. Written and verbal consent were obtained from all participants. Human subject research approval was obtained from the VINMEC Healthcare System and Vanderbilt University Medical Center (VUMC).

Information on demographic characteristics, health status, medication history, and dietary habits was collected through in-person interviews using a structured questionnaire at study enrollment and after the 16-week trial by trained interviewers from the Vietnam Colorectal Cancer and Polyps Research (VinCAPR) team. Additionally, 24 h dietary recalls (24-HDRs) were utilized to capture dietary intake three days prior to stool collection. The consumption per day of each food group was calculated, including grain, staple foods, fruits, vegetables, peanuts, legumes, red meats, poultry, fish and shrimp, eggs, dairy products, and soybean products. Furthermore, compliance with the peanut intervention was assessed through a self-reported monthly survey conducted via telephone calls 3 times for 16 weeks (Figure 1). We captured and managed the in-person surveys using the Research Electronic Data Capture (REDCap) data management platform hosted at the VINMEC Healthcare System.

In this current analysis, we excluded non-compliant participants (n = 10) and those who received antibiotic treatment during the seven days prior to stool collection (n = 12) at the end of the trial. Finally, 35 participants from the peanut intervention group and 43 from the control group were included in the analysis.

### 2.2. Stool Sample Collection

Participants’ stool samples were collected twice using fecal occult blood test (FOBT) cards, first at the beginning and then again on the last date of the trial (after 16 weeks of study, following a standard protocol). Each time, trained study staff provided three stool collection cards and three Fe-Col^®^ stool collection paper bands for each participant and clear instructions on collecting stool samples at home. Within six hours of the collection, the stool samples were transferred in a cool box to the VinCAPR research laboratory and stored in a freezer at −80 °C.

### 2.3. Microbiome Profiling

#### 2.3.1. DNA Extraction and Shotgun Metagenomic Sequencing

We used the DNeasy PowerSoil Pro Kit (Qiagen Cat. No. 47014, Hilden, Germany) to isolate microbial genomic DNA from stool samples, following the manufacturer’s protocols. For shotgun metagenomic sequencing, the DNBSEQ Short-read library preparation kit was utilized to build sequencing libraries from DNA samples, and sequencing was conducted at paired-end 150 bp using the MGISEQ-2000 at BGI Americas (Cambridge, MA, USA). DNA extraction, library preparation, and sample sequencing were performed in one batch. The quality values of Q20 (percentage of bases whose base call accuracy exceeds 99%) and the GC content were evaluated, with an average of 97.3% and 45.1%, respectively.

#### 2.3.2. Sequencing Data Processing

On average, 20.01 million (Min–Max: 16.98–20.16 million) raw sequencing reads were obtained for each sample from our study. We used Trimmomatic v0.39 to process raw reads and trim low-quality bases. Reads with fewer than 105 nucleotides (i.e., 70% of original read lengths) were discarded [33]. Next, Bowtie2 v2.3.0 was used to eliminate reads that could be mapped on the human genome (GRCh38) [34]. Then, the remaining clean reads were subjected to taxonomic profiling and estimation of the absolute abundance of microbial taxa using Kraken v2.1.1 and Bracken v2.6. We utilized bacterial genomes from the Unified Human Gastrointestinal Genome (UHGG) as a reference [35,36,37]. Within each sample, only taxa with a relative abundance of ≥0.00001 were considered detected [38,39]. Finally, HUMAnN 3.0 was used to perform functional profiling of the gut microbiome and estimate the relative abundance of gut microbial metabolic pathways [40]. We utilized the UniRef90 comprehensive protein database as a reference.

### 2.4. Statistical Analysis

We first evaluated the impact of peanut intervention with changes in overall microbial diversity, including α-diversity and β-diversity, and the overall stability of human gut microbiota. As both the α- and β-diversity of species estimates might be affected by sequencing depth [41], we rarefied the species-level absolute abundance, i.e., read counts, of every sample to the minimum number of clean reads (n = 15,160,410) among 156 samples, using the R function vegan::rarefy [42]. Next, we estimated α-diversity and β-diversity based on the rarefied species-level absolute abundance data and relative abundance data of gut microbial metabolic pathways using the R functions vegan::diversity and vegan::vegdist, respectively [42]. We measured α-diversity by the Chao1 richness index (Chao1 index), Shannon–Wiener diversity index (Shannon index), and Pielou evenness index (Pielou index). Differences across groups (control vs. peanut intervention) and time (baseline vs. 16-week follow-up) were compared using Wilcoxon rank sum tests. β-diversity was measured by the Bray–Curtis dissimilarity matrix (Bray–Curtis) and Jaccard dissimilarity matrix (Jaccard). The Permutational Multivariate Analysis of Variance (PERMANOVA) test was applied to assess whether there was a difference in β-diversity by study group and time [43] with 999 permutations using the R functions vegan [42]. Finally, we examined the associations of peanut intervention with changes in the Chao1, Shannon, Pielou, Bray–Curtis, and Jaccard indexes using linear regression models via the R package “MicrobiomeStat” [44]. Covariates included in the models include age (years), gender (male/female), body mass index (BMI; kg/m^2^), poultry intake, and other legume intake, with the exclusion of peanut consumption (g/day). To determine the overall stability of human gut microbiota from baseline to 16 weeks post-intervention, we calculated intraindividual differences (beta diversity distance within-sample) and subtracted them from 1. We used the formula 1 – Bray–Curtis Index within-sample (baseline vs. 16-week follow-up) to determine species stability and the stability of gut microbial metabolic pathways. Stability data were continuous probability distributions set on the interval [0, 1], i.e., beta distribution. A stability of 1 indicated individuals had identical samples across time points, whereas a stability of 0 meant they had completely different samples [45]. Beta regression analysis was performed using the R package “*betareg*” [46]. β coefficients, standard error (SE), and *p* values were calculated in models with adjustment for the aforementioned covariates. The statistical analyses were performed with two-sided tests. *p* values < 0.05 were considered statistically significant.

Secondly, we evaluated the association of peanut intervention with individual gut microbial taxa from phylum to species. We limited our analysis to those microbial taxa that were present in >10% of samples and had a median relative abundance > 0.001%. Linear mixed-effects models (LMMs) for differential abundance analysis (LinDA) were applied with adjustment for age, gender, BMI, and other legume intake via the R package “MicrobiomeStat” v.1.1.3 [44]. LinDA-LMM used the centered log-ratio (clr) transformation to normalize the absolute abundance of taxa at each taxonomic level from phylum to species, with zeros replaced by the next minimal read count value of the whole dataset [47]. β coefficients, SE, and *p* values for individual microbial taxa were produced. The false discovery rate (FDR) was calculated at each taxonomic level for multiple testing sets. The same approach was applied to evaluate peanut intervention with individual microbial metabolic pathways. We confined our analysis to those microbial metabolic pathways observed in >10% of samples and median relative abundance >0.001%. All associations with FDR-corrected *p*-values of <0.1 were considered statistically significant.

We also examined the impact of peanut intervention on gut microbiome sub-community structure. Latent Dirichlet Allocation (LDA), a three-level Bayesian probabilistic generative model that reveals latent structures in unlabeled data, Ref. [48] was utilized to infer sub-communities in gut microbiota. This unsupervised machine learning method determines the microbiota subgroups by the taxa distribution in the observations [49,50,51]. After removing taxa that were detected in <20% of all samples and had a median relative abundance of <0.1%, absolute abundance data of 321 species were included in the LDA analyses using the R “topicmodels” package (v0.2-16). In our study, the gut microbiota was assigned to 45 species-level subgroups (Supplementary Figure S1). The number of subgroups was determined following a recently described strategy [52]. Briefly, we fitted a series of LDA models with the number of subgroups ranging from 3 to 99, plotted model performance against the number of subgroups, and determined the optimal subgroup number according to the first jump in model performance [52]. Given a specific number of subgroups, the LDA algorithm estimates the probability that a microbial taxon belongs to each subgroup and the probability that each subgroup belongs to a participant [52]. The probability values of all subgroups for a participant and all taxa for a subgroup add up to 1. We estimated the abundance of data on species-level subgroups and evaluated the associations of gut microbial taxa with peanut intervention for species-level subgroups using linear mixed-effects models.

Finally, we used the Bray–Curtis metric to calculate the stability of 321 individual species and 300 metabolic pathways, which were present in >20% of samples and had a median relative abundance of >0.01%, via comparison of the absolute abundance/relative abundance of each species/metabolic pathway across all participants at baseline and post-16 weeks. We applied beta regression analysis to estimate the β coefficients, standard error (SE), *p* values, and FDR of each single feature in association with peanut intervention. The R packages “MicrobiomeStat” and “MicrobiotaProces” were used for visualizations of microbiome data [53]. All statistical analyses were performed using R version 4.3.2.

## 3. Results

The mean age of study participants was 54.9 (SD: 8.6) years for the peanut intervention group and 56.2 (SD: 9.0) years for the control group. Compared with controls, participants in the peanut intervention group were similar regarding gender, age groups, and BMI levels. No differences were observed between peanut intervention and control groups concerning self-report health issues during the three months before trial enrollment, including diabetes, hypertension, hyperlipidemia, infection requiring antibiotic treatment, food allergy, and health issues that changed diet habits or bowel movements (Supplementary Table S1).

The peanut intervention group and the control group had no significant difference in intake of grain, staples, fruits, vegetables, legumes (bean and peas), red meat, poultry, fish and shrimp, eggs, dairy products, soybean products, and other nuts (e.g., walnuts, lotus seeds, or pumpkin seeds) at baseline and the end of the trial. Likewise, except for poultry intake among controls, no significant differences were found in the consumption of food groups within the intervention and the control groups, both before and after the end-intervention. The controls had a higher intake of poultry at the end of the trial than at baseline (Supplementary Table S2).

**Figure 2:**
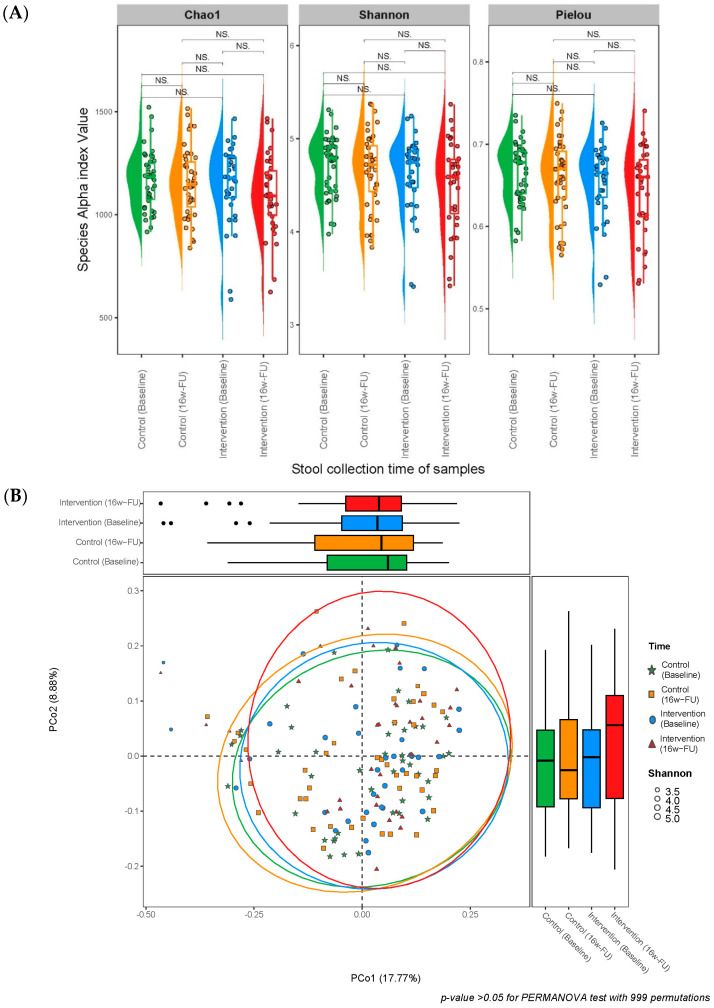

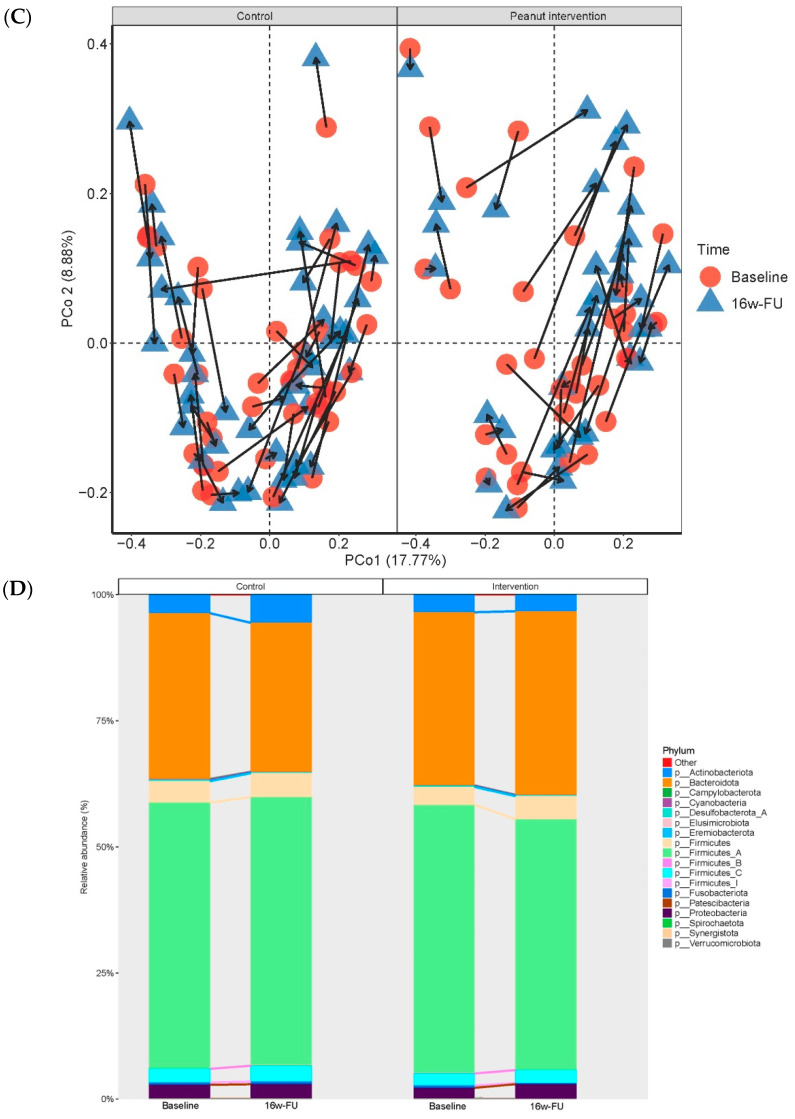
(**A**) Alpha diversity at the species level (measured in terms of the Chao1 richness, Shannon-Wiener diversity, and Pielou indexes) over time. (**B**) Principal coordinate analysis (PCoA) of Bray–Curtis diversity over time. (**C**) Beta diversity changes within individual subjects over time by the trial groups in principal coordinate analysis (PCoA) of Bray–Curtis diversity. (**D**) Relative abundance by study group over time. Abbreviation: 16w–FU: 16-week follow-up; NS.: Non-significant.

No significant differences (all *p* > 0.05) were found in α-diversities (Chao1, Shannon, and Pielou indexes) at a species or metabolic pathway level within and between the intervention and control groups, between pre- and post-intervention (Table 1 and Figure 2A). The mean stability of gut microbial species and metabolic pathways was 0.575 and 0.899 for the peanut intervention group and 0.600 and 0.890 for the control group, respectively. No significant differences were observed in the overall stability of species and metabolic pathways between the intervention and control groups (Table 1). Similarly, no significant difference in the Bray–Curtis dissimilarity matrix was found between the control and peanut intervention groups (*p* > 0.05 for the PERMANNOVA test with 999 permutations; Figure 2B). Beta diversity changes within individual subjects over time were not significant in the study groups (Figure 2C).

Multivariable regression analyses showed that peanut intervention was not associated with changes in α- and β-diversities and overall stability of gut microbial species and metabolic pathways (all *p* > 0.05; Table 2).

Among the 156 stool samples from 78 participants, a total of 20 phyla, 28 classes, 67 orders, 204 families, 1078 genera, and 3678 species were identified. At the phylum level, the gut microbiota had high proportions (i.e., relative abundance) of *Actinobacteriota*, *Bacteroidota*, and *Firmicutes* group*s*, with a dominance of *Firmicutes*, *Firmicutes* A and *Firmicutes* C, *Proteobacteria*, and a smaller proportion of other phyla, including *Campylobacterota*, *Cyanobacteria*, *Desulfobacterota* A, *Elusimicrobiota*, *Fusobacteriota*, *Patescibacteria*, *Spirochaetota*, *Synergistota*, and *Verrucomicrobiota* (Figure 2D). After excluding microbial taxa with a prevalence of <10% in all samples and median relative abundance <0.001%, a total of 13 phyla, 14 classes, 37 orders, 81 families, 330 genera, and 988 species were included to evaluate the association of peanut intervention with individual microbial taxa.

Covariate-adjusted linear mixed-effect models showed that peanut intervention was significantly associated with a reduced abundance of 12 microbial taxa, including five phyla, five classes, and two orders after FDR correction (FDR < 0.1). We found that peanut intervention was significantly associated with a reduced abundance of phylum *Actinobacteriota*, with a β coefficient of −0.915 (SE: 0.315; *p* = 0.004 and FDR = 0.035), which was driven by its class *Coriobacteriia* (β [SE] = −1.041 [0.222]; *p* = 6.58 × 10^−6^; FDR = 9.22 × 10^−5^) and order *Coriobacteriales* (β [SE] = −0.991 [0.243]; *p* = 7.56 × 10^−5^; FDR = 0.003). The phylum *Firmicutes* (β [SE] = −0.685 [0.242]; *p* = 0.005; FDR = 0.035) and its class *Bacilli* (β [SE] = −0.690 [0.228]; *p* = 0.003; FDR = 0.020) and order *Erysipelotrichales* (β [SE] = −0.799 [0.243]; *p* = 1.25 × 10^−3^; FDR = 0.023) were less abundant after peanut intervention. Additionally, peanut intervention was significantly associated with a reduced abundance of phylum *Firmicutes* A, with a β (SE) of −0.314 (0.137); *p* = 0.024 and FDR = 0.065, which was driven by its class *Clostridia* (β [SE] = −0.318 [0.126]; *p* = 0.012; FDR = 0.053). Last but not least, the abundance of three phyla, including *Proteobacteria* and *Synergistota*, was significantly decreased after peanut intervention, with a respective β of −0.814 (SE: 0.341; *p* = 0.018 and FDR = 0.065) and −0.762 (SE: 0.336; *p* = 0.025; FDR = 0.065), which was driven by their classes, including class *Gammaproteobacteria* (β [SE] = −0.861 [0.350]; *p* = 0.015; FDR = 0.053) and class *Synergistia* (β [SE] = −0.769 [0.336]; *p* = 0.023; FDR = 0.066), respectively (Table 3). Changes in the relative abundance of these taxa between 16-week follow-up and baseline were shown in Supplementary Table S3 and Supplementary Figure S2.

The gut microbiome community was classified into 45 heterogeneous subgroups at the species level. Six species-level subgroups had a dominant species with an occurrence probability of >50%, including 72.58% for *Prevotella copri* in subgroup 29, 68.78% for *Bacteroides* A *plebeius* A in subgroup 31, 66.15% for *Bifidobacterium pseudocatenulatum* in subgroup 15, 63.50% for *Escherichia coli* D in subgroup 21, 54.20% for *Ruminococcus* E *bromii* B in subgroup 26, and 53.34% for *MGYG-HGUT-00200* in subgroup 1 (Supplementary Table S4). Peanut intervention was significantly associated with a lower abundance of seven species-level subcommunities after FDR correction (FDR < 0.1), with *MGYG-HGUT-00200* (53.34% in subgroup 1), *Roseburia inulinivorans* (24.12% in subgroup 7), *Bifidobacterium pseudocatenulatum* (66.15% in subgroup 15), *Escherichia coli* D (63.50% in subgroup 21), *Ruminococcus* D *bicirculans* (26.69% in subgroup 24), and *Holdemanella biformis* (30.75% in subgroup 35) being the top representative taxa. The β of each (SE; *p*-value; FDR) was −4.633 (1.415; *p* = 1.33 × 10^−3^; FDR = 0.020), −6.125 (1.804; *p* = 8.85 × 10^−4^; FDR = 0.020), −2.838 (1.085; *p* = 9.84 × 10^−3^; FDR = 0.074), −4.664 (1.351; *p* = 7.23 × 10^−4^; FDR = 0.020), −4.910 (1.733; *p* = 5.26 × 10^−4^; FDR = 0.059), and −4.001 (1.450; *p* = 6.56 × 10^−3^; FDR = 0.059) for the species-level subcommunities 1, 7, 15, 21, 24, and 35, respectively (Table 4).

A total of 515 gut microbial metabolic pathways were identified, and their relative abundances were estimated among 156 samples. After excluding microbial metabolic pathways that were present in <10% of samples and had a median of relative abundance <0.001%, a total of 366 metabolic pathways were included in the evaluation of the association of peanut intervention with individual metabolic pathways. Multivariable linear mix-effect regression analysis showed that peanut intervention was significantly associated with reduced enrichment of 24 gut microbial metabolic pathways after FDR correction (FDR < 0.1). The relative abundances of eight microbial metabolic pathways involved in the superpathways menaquinol-8 biosynthesis I (PWY-5838); menaquinol-7 biosynthesis (PWY-5840); menaquinol-9 biosynthesis (PWY-5845); demethylmenaquinol-8 biosynthesis I (PWY-5861); demethylmenaquinol-9 biosynthesis (PWY-5862); menaquinol-11 biosynthesis (PWY-5897); menaquinol-12 biosynthesis (PWY-5898); and menaquinol-13 biosynthesis (PWY-5899) were significantly reduced after peanut intervention, with a β (SE; *p*-value) of −0.689 (0.183; *p* = 2.49 × 10^−4^; FDR = 0.017); −0.737 (0.233; *p* = 0.002; FDR = 0.037); −0.639 (0.194; *p* = 1.25 × 10^−3^; FDR = 0.028); −0.739 (0.196; 2.34 × 10^−4^; FDR = 0.017); −0.679 (0.204; *p* = 1.09 × 10^−3^; FDR = 0.028); and −0.704 (0.193; *p* = 3.62 × 10^−4^; FDR = 0.017) for PWY-5897, PWY-5898, and PWY-5899, respectively. Additionally, peanut intervention was significantly inversely associated with the Allantoin degradation to glyoxylate III (PWY-5705), L-ascorbate degradation I and II (bacterial, aerobic; PWY-6961 and PWY0-301) and Phytol degradation (PWY66-389) pathways, with a respective β (SE; *p*-value) of −1.454 (0.481; *p* = 0.003; FDR = 0.050); −1.009 (0.361; *p* = 0.006; FDR = 0.087), −0.937 (0.310; *p* = 0.003; FDR = 0.050), and −1.825 (0.559; *p* = 1.36 × 10^−3^; FDR = 0.028) (Table 5). Changes in the relative abundance of these gut microbial metabolic pathways between 16-week follow-up and baseline are shown in Supplementary Table S5.

Peanut intervention was significantly associated with the increased stability of eight species belonging to members of the phylum *Firmicutes* A, such as *Oscillibacter* sp900066435 (β [SE] = 0.986 [0.273]; *p* = 2.99 × 10^−3^; FDR = 0.029), *Faecalibacterium prausnitzii* F (β [SE] = 0.901 [0.274]; *p* = 1.02 × 10^−3^; FDR = 0.048), and *Faecalibacterium prausnitzii* H (β [SE] = 0.686 [0.226]; *p* = 0.002; FDR = 0.070), as well as three microbial metabolic pathway involved in nitrate reduction V (PWY-5675; β [SE] = 1.040 [0.306]; *p* = 6.73 × 10^−4^; FDR = 0.068). Conversely, peanut intervention was significantly associated with the decreased stability of the species *Alistipes putredinis* (β [SE] = −1.065 [0.299]; *p* = 3.67 × 10^−4^; FDR = 0.029) and three metabolic pathways including L-lysine biosynthesis III (PWY-2942; β [SE] = −0.563 [0.172]; *p* = 1.03 × 10^−3^; FDR = 0.078), the superpathway of guanosine nucleotide degradation (PWY-6595; β [SE] = −0.982 [0.287]; *p* = 6.27 × 10^−4^; FDR = 0.068), and guanosine nucleotide degradation I (PWY-6607; −0.976 [0.287]; *p* = 6.77 × 10^−4^; FDR = 0.068) (Table 6).

## 4. Discussion

In the Vietnam Peanut Trial Study, we found that changes in gut microbial diversities and the overall stability of gut microbial species and metabolic pathways were minor after a 16-week peanut intervention. However, this peanut intervention led to reduced enrichment of five phyla, including *Actinobacteriota*, *Firmicutes*, *Firmicutes* A, *Proteobacteria*, and *Synergistota*, as well as five classes and two orders belonging to these phyla. Additionally, peanut intervention was significantly and inversely associated with the abundance of species-level sub-communities, with a dominant representation of the species *Bifidobacterium pseudocatenulatum*, *Escherichia coli* D, *Holdemanella biformis*, *Ruminococcus* D *bicirculans*, *Roseburia inulinivorans*, and MGYG-HGUT-00200. Moreover, the relative abundances of 24 microbial metabolic pathways, particularly those involving biosynthesis of menaquinone and demethylmenaquinone, were significantly reduced after peanut intervention. Finally, peanut intervention was significantly associated with the increased stability of several species, such as *Faecalibacterium prausnitzii* F and H, as well as a microbial metabolic pathway involved in nitrate reduction V, which may play an important role in human health, especially cardiovascular health [54,55,56].

Few studies have investigated the influence of peanut consumption on the gut microbiome. Animal studies suggested that both peanuts and their by-products may have beneficial impacts on metabolic syndromes and their risk factors, such as body weight, glucose, and lipid metabolism, by regulating the gut microbiota, even though gut microbiota results were inconsistent [16]. In human studies, our previous study conducted among 2302 elderly Chinese adults from two large cohort studies found that long-term peanut consumption was significantly associated with a higher α-diversity index (i.e., Chao1 index) among Chinese men but a lower α-diversity index, including Shannon and Simpson indexes, among Chinese women [57]. Long-term peanut consumption was also significantly associated with an increased abundance of the order *Actinomycetales* and an unclassified genus of the family *Enterobacteriaceae* [57]. Moreover, a 12-week randomized clinical trial was conducted on 209 adults with metabolic syndrome risk, in which 109 participants in the intervention group received 56 g of peanuts and 100 of those in the control group received 82 g of rice bars per day [29]. Overall, this study found that the gut microbiota diversity and composition among adults with metabolic syndrome risk had minor changes after peanut intervention; the abundance of three genera, including *Bilophila*, *Coprococcus*_3, and *Dorea*, decreased after peanut intervention [29]. Similarly, a randomized, crossover trial conducted on 50 adults with 28 g/d of dry-roasted, unsalted peanuts for six weeks with a four-week washout period found no differences in gut microbiota diversity but a significantly increased abundance of *Ruminococcaceae* [30].

Our 16-week peanut intervention did not lead to significant changes in gut microbial diversity, which is consistent with findings from most previous studies on peanuts and other types of nuts, showing that nut consumption had little or no effect on gut microbial diversity [27,29,30]. Our findings also agree with earlier observations that short-term feeding trials of nuts, including tree and ground nuts, influenced the gut microbial composition more than the overall gut microbial diversity [27]. We observed that peanut intervention significantly reduced the abundance of five phyla, including *Actinobacteriota*, *Firmicutes*, *Firmicutes* A, *Proteobacteria*, and *Synergistota*, and several classes and orders within these phyla, such as classes *Coriobacteriia*, *Bacilli*, *Clostridia*, *Gammaproteobacteria*, and *Synergistia*, as well as orders *Coriobacteriales* and *Erysipelotrichales* after FDR correction (FDR < 0.1). The changes in individual gut microbial taxa corresponded to the alterations in gut microbiome sub-community structure. The peanut intervention was significantly associated with a lower abundance of six species-level sub-communities, with a dominant representation of species *Bifidobacterium pseudocatenulatum* (within phylum *Actinobacteriota*), *Escherichia coli* D (within class *Gammaproteobacteria*), *Holdemanella biformis* (within order *Erysipelotrichales*), *Ruminococcus* D *bicirculans*, *Roseburia inulinivorans*, and MGYG-HGUT-00200 (three members of class *Clostridia*).

Peanut intervention tended to increase the abundance of members of the family *Ruminococcaceae*, such as species *Faecalibacterium prausnitzii* E, J, and K, *Faecalibacterium* sp003449675, and *Gemmiger formicilis*, as well as members of the family *Bacteroidaceae* such as species *Bacteroides* A sp000434735, *Paraprevotella xylaniphila*, and *Prevotella*, and genera and species including *Prevotella oris*, *Prevotella* sp000431975, *Prevotella* sp000434515, and *Prevotella* sp003447235 (*p*-value < 0.05, all FDR > 0.1). A pediatric study found that children with peanut allergies had a lower abundance of *Prevotella* species in their gut microbiota, whereas *Prevotella* species were overrepresented in non-allergic controls [58]. Some studies linked the species *Prevotella* with health benefits such as reduced visceral fat and improved glucose metabolism [59,60]. In contrast, others reported the involvement of the species *Prevotella* with metabolic syndrome, insulin resistance, hypertension, obesity, persistent gut inflammation, and rheumatoid arthritis [61]. At a *p*-value of <0.05, peanut intervention also tended to decrease the abundance of 80 out of 122 microbial taxa (65.6%), particularly members of the genus *Escherichia*, including *E. albertii*, *E. coli* D, *E. fergusonii*, *E. marmotae*, and *E.* sp000208585all (FDR >0.1). Moreover, the relative abundances of microbial metabolic pathways involving biosynthesis of menaquinol-7, -8, -9, -11, -12, and -13, as well as demethylmenaquinol-8 and -9 biosynthesis, were significantly reduced after peanut intervention (FDR <0.1). The species of the genus *Escherichia* are known for producing bacteria-derived menaquinones, mainly menaquinone-8 (Vitamin K), [62] which support the growth of neighboring gut bacteria in the human gut microbiome [63]. We speculated that a reduction in bacteria-derived menaquinones due to short-term peanut intervention could decrease the presence of menaquinone in the human gut, which would then affect the growth of several species, including beneficial and harmful gut bacteria. This could be a “shock effect”—a temporary reduction in gut microbial composition due to a rapid diet change such as a short-term peanut feeding trial [27]. However, the temporary reduction in gut microbial composition observed in our study did not significantly impact the overall diversity and stability of gut microbiota. Furthermore, the dosage of a 16-week peanut intervention, with 50 g of peanut per day, may not be sufficient to modulate the proliferation of microbes in the human gut. More studies are needed to elucidate this hypothesis, with consideration for a higher dose and a more extended intervention period.

Although the 16-week peanut intervention did not change the overall stability of gut microbial species and metabolic pathways, it did increase the stability of several species including *Faecalibacterium prausnitzii* F, *Faecalibacterium prausnitzii* H, and *Oscillibacter* sp900066435 (FDR < 0.1). *F. prausnitzii* has been known as an acetate consumer that mainly produces the SCFA butyrate and bioactive anti-inflammatory molecules (e.g., shikimic and salicylic acids) [64,65] and is recognized as a key player in the gut health and physiology of the host [66]. Butyrate, a major energy source for the colonic epithelial cells, is essential for maintaining colonic mucosal health [67]. Butyrate also plays a regulatory role in transepithelial fluid transport, preventing and inhibiting colonic carcinogenesis and improving mucosal inflammation, oxidative status, epithelial defense barrier, and modulation of visceral sensitivity and intestinal motility [68]. Additionally, peanut intervention led to an increase in the stability of a microbial metabolic pathway involved in nitrate reduction V. Nitrate from the diet may be converted to nitrite and then to other reduced nitrogen biomolecules such as nitric oxide, ammonia, urea, and possibly nitrogen gas by gut bacteria. The conversion may induce vasodilation and decrease blood pressure [55], thereby lowering the risk of cardiovascular disease, myocardial infarction, and stroke [56]. However, the influence of our peanut interventions on the stability of the microbial metabolic pathway involved in nitrate reduction V was unclear due to its inverse association with the abundance of this metabolic pathway (FDR < 0.1). Further research into the link between symbiotic bacteria, nitrogen oxide metabolism, and human health is needed.

Our study is the longest peanut trial on the gut microbiome (i.e., a 16-week peanut intervention) to date. We applied shotgun metagenomic sequencing and used a human bacterial genome from the UHGG collection as a reference. UHGG is a massive sequence catalog containing information on ~4600 species, with 71% lacking a cultured representative [35,36,37]. This allowed us to estimate the prevalence and abundance of microbial taxa with enhanced resolution and accuracy. In addition to evaluating individual microbial taxa, we utilized LDA analysis to assess the alterations of the gut microbiome community in association with the peanut intervention. However, the underlying structure of the gut community cannot be resolved. There are other limitations to consider when interpreting our findings. First, our randomized clinical trial had a small sample size, compromising the statistical power to capture the weak-to-modest associations between peanut intervention and gut microbiome change. Another limitation is non-compliance in the peanut intervention group due to diminished participants’ commitment and the lengthy trial duration. Additionally, “wash-out” fecal samples after the end of the trial were not collected and analyzed in this study, which prevented us from investigating whether the gut microbiome would revert back to pre-trial status. Moreover, our findings may not be generalizable to the changes in gut microbiome profile resulting from long-term peanut consumption. Finally, the direct impact of peanut consumption and gut microbiome on health-related outcomes was not evaluated in our study. However, our study is the first step toward a better understanding of the influence of peanut consumption on gut microbiome change. The findings provide a strong justification to conduct a larger and longer intervention study to investigate the direct impact of gut-microbiome-mediated health effects of peanut consumption.

## 5. Conclusions

In summary, a 16-week peanut intervention led to a lower abundance of several gut microbial taxa, metabolic pathways, and species-level sub-communities, with a dominant representation of species *Bifidobacterium pseudocatenulatum*, *Escherichia coli* D, *Holdemanella biformis*, *Ruminococcus* D *bicirculans*, *Roseburia inulinivorans*, and MGYG-HGUT-00200. The peanut intervention also increased the short-term stability of bacteria such as *F. prausnitzii* and a microbial metabolic pathway involved in nitrate reduction V. Further research is needed to confirm these findings and investigate the direct impact of gut-microbiome-mediated health effects, particularly on cardiovascular health, from peanut consumption.

## Figures and Tables

**Table 1:** Alpha diversity and overall species/pathway stability by study group.

	Control	Peanut Intervention	*p* _for Intervention vs. Control_
	n = 43	n = 35	
	Mean ± SD	Mean ± SD	
Alpha diversity			
Chao1 index			
Species Chao1 index at baseline	1167 ± 131	1146 ± 190	0.960
Species Chao1 index at 16w-FU	1152 ± 166	1100 ± 190	0.263
*p* _for baseline vs. FU_	0.592	0.145	
Pathways Chao1 index at baseline	381 ± 35	378 ± 40	0.872
Pathways Chao1 index at 16w-FU	379 ± 39	382 ± 30	0.924
*p* _for baseline vs. FU_	0.938	0.907	
Shannon index			
Species Shannon index at baseline	4.708 ± 0.316	4.613 ± 0.418	0.363
Species Shannon index at 16w-FU	4.656 ± 0.405	4.509 ± 0.473	0.179
*p* _for baseline vs. FU_	0.547	0.256	
Pathways Shannon index at baseline	5.185 ± 0.159	5.168 ± 0.138	0.697
Pathways Shannon index at 16w-FU	5.194 ± 0.166	5.175 ± 0.160	0.617
*p* _for baseline vs. FU_	0.898	0.879	
Pielou’s evenness			
Species Pielou index at baseline	0.667 ± 0.036	0.655 ± 0.045	0.303
Species Pielou index at 16w-FU	0.661 ± 0.046	0.644 ± 0.053	0.233
*p* _for baseline vs. FU_	0.530	0.427	
Pathways Pielou index at baseline	0.873 ± 0.019	0.872 ± 0.0166	0.976
Pathways Pielou index at 16w-FU	0.876 ± 0.020	0.871 ± 0.021	0.152
*p* _for baseline vs. FU_	0.497	0.462	
Stability (1 − Intraindividual difference) ^a^			
Species stability	0.575 ± 0.122	0.600 ± 0.103	0.435
Pathways stability	0.899 ± 0.045	0.890 ± 0.049	0.289

^a^ Stability: 1 − Bray–Curtis Index _within-sample (Baseline vs. FU)_; Individuals with a stability of 1 have identical samples across time points, while a stability of 0 meant the samples were nothing alike.

**Table 2:** Association of peanut intervention with changes in microbiome diversity (n = 78).

Variable	Species			Pathways		
	β	SE	*p*	β	SE	*p*
Alpha diversity						
Chao1 index	−0.024	0.041	0.555	0.037	0.036	0.307
Shannon index	−0.008	0.027	0.778	−0.001	0.010	0.906
Pielou index	−0.004	0.023	0.846	−0.008	0.008	0.356
Beta diversity						
Bray–Curtis distance	−0.023	0.018	0.203	0.011	0.007	0.142
Jaccard Index distance	−0.021	0.018	0.229	0.017	0.011	0.135
Overall stability	0.105	0.100	0.294	−0.112	0.093	0.229

**Table 3:** Association of peanut intervention with individual microbial taxa *.

Microbial Taxa	Average RA, Median (%)|Pre (%)		β (SE)	*p*	FDR
	Control	Peanut Intervention			
	(n = 43)	(n = 35)			
Phylum *Actinobacteriota*	2.8|100	2.3|100	−0.915 (0.315)	0.004	0.035
Class *Coriobacteriia*	2.1|100	1.8|100	−1.041 (0.222)	6.58 × 10^−6^	9.22 × 10^−5^
Order *Coriobacteriales*	2.1|100	1.8|100	−0.991 (0.243)	7.56 × 10^−5^	0.003
Phylum *Firmicutes*	3.3|100	2.8|100	−0.685 (0.242)	0.005	0.035
Class *Bacilli*	3.3|100	2.8|100	−0.690 (0.228)	0.003	0.020
Order *Erysipelotrichales*	1.77|100	1.6|100	−0.799 (0.243)	1.25 × 10^−3^	0.023
Phylum *Firmicutes A*	54.4|100	50.0|100	−0.314 (0.137)	0.024	0.065
Class *Clostridia*	54.4|100	50.0|100	−0.318 (0.126)	0.012	0.053
Phylum *Proteobacteria*	1.4|100	1.3|100	−0.814 (0.341)	0.018	0.065
Class *Gammaproteobacteria*	1.4|100	1.3|100	−0.861 (0.350)	0.015	0.053
Phylum *Synergistota*	1.18 × 10^−3^|57.0	1.29 × 10^−3^|57.1	−0.762 (0.336)	0.025	0.065
Class *Synergistia*	1.18 × 10^−3^|57.0	1.29 × 10^−3^|57.1	−0.769 (0.336)	0.023	0.066

* A LinDA-linear mixed-effect model was conducted for the absolute abundance of microbial taxa with adjustment for age, gender, BMI, and the intake of poultry and legumes. The false discovery rate (FDR) was calculated at each taxonomic level. We limited our analysis to 13 phyla (20), 14 classes (28), 37 orders (67), 81 families (204), 330 gena (1078), and 988 species (3678) with a prevalence > 10% and a median of relative abundance > 0.001%. Abbreviation: RA: relative abundance; FDR: false discovery rates; Pre: prevalence.

**Table 4:** Association of peanut intervention with species-levels subgroups 1,7, 15, 21, 24, and 35 *.

Subgroup and Top 5 Taxa	β (SE)	*p*	FDR
Subgroup1	−4.633 (1.415)	1.33 × 10^−3^	0.020
Species MGYG-HGUT-00200 (53.34%)			
Species *Faecalicatena faecis* (11.75%)			
Species *Anaerostipes hadrus* (2.94%)			
Species *Blautia* A sp900066165 (2.56%)			
Species MGYG-HGUT-02772 (1.62%)			
Subgroup 7	−6.125 (1.804)	8.85 × 10^−4^	0.020
Species *Roseburia inulinivorans* (24.12%)			
Species *Bacteroides* B *dorei* (4.33%)			
Species *Faecalicatena torques* (3.93%)			
Species *Holdemanella* sp002299315 (3.71%)			
Species *Dorea longicatena* B (3.34%)			
Subgroup 15	−2.838 (1.085)	9.84 × 10^−3^	0.074
Species *Bifidobacterium pseudocatenulatum* (66.15%)			
Species *Anaerostipes hadrus* (6.14%)			
Species *Fusicatenibacter saccharivorans* (3.62%)			
Species *Dorea longicatena* B (3.12%)			
Species *Faecalibacterium prausnitzii* D (1.80%)			
Subgroup 21	−4.664 (1.351)	7.23 × 10^−4^	0.020
Species *Escherichia coli* D (63.50%)			
Species *Escherichia fergusonii* (6.90%)			
Species *Escherichia* sp000208585 (3.76%)			
Species *Bacteroides stercoris* (2.37%)			
Species *Lachnospira rogosae* (1.95%)			
Subgroup 24	−4.910 (1.733)	5.26 × 10^−3^	0.059
Species *Ruminococcus* D *bicirculans* (26.69%)			
Species *Faecalibacterium prausnitzii* D (11.07%)			
Species *Fusicatenibacter saccharivorans* (10.02%)			
Species *Blautia* A sp900066165 (4.28%)			
Species *Bacteroides stercoris* (3.45%)			
Subgroup 35	−4.001 (1.450)	6.56 × 10^−3^	0.059
Species *Holdemanella biformis* (30.75%)			
Species *Blautia* A *wexlerae* (8.45%)			
Species *Holdemanella* sp002299315 (3.05%)			
Species *Dorea formicigenerans* (2.76%)			
Species *Dorea longicatena* B (2.51%)			

* A LinDA-linear mixed-effect model was conducted with adjustment for age, gender, BMI, and intake of poultry and legumes. FDR was calculated by the 45 species-level subgroups.

**Table 5:** Association of peanut intervention with individual microbial metabolic pathways *.

	Microbial Metabolic Pathways	Average RA, Median|Pre (%)		β (SE)	*p*	FDR
		Control	Intervention			
		(n = 43)	(n = 35)			
NAD-BIOSYNTHESIS-II	NAD salvage pathway III (to nicotinamide riboside)	0.0135|95.3	0.0106|94.3	−1.279 (0.35)	3.52 × 10^−4^	0.017
P461-PWY	Hexitol fermentation to lactate, formate, ethanol and acetate	0.0895|100	0.0801|100	−0.507 (0.141)	4.47 × 10^−4^	0.018
P4-PWY	Superpathway of L-lysine, L-threonine and L-methionine biosynthesis I	0.0611|100	0.0578|97.1	−0.757 (0.184)	6.63 × 10^−5^	0.012
PWY0-301	L-ascorbate degradation I (bacterial, anaerobic)	0.0149|94.2	0.0189|94.3	−1.009 (0.361)	0.006	0.087
PWY0-781	Aspartate superpathway	0.0644|100	0.0609|97.1	−0.752 (0.183)	6.54 × 10^−5^	0.012
PWY-5675	Nitrate reduction V (assimilatory)	0.0107|89.5	0.0157|92.9	−1.27 (0.389)	1.35 × 10^−3^	0.028
PWY-5705	Allantoin degradation to glyoxylate III	0.0016|57	0.0017|60	−1.454 (0.481)	0.003	0.050
PWY-5723	Rubisco shunt	0.0251|96.5	0.0258|94.3	−1.225 (0.37)	1.18 × 10^−3^	0.028
PWY-5837	2-carboxy-1,4-naphthoquinol biosynthesis	0.0241|96.5	0.0207|100	−0.824 (0.233)	5.31 × 10^−4^	0.019
PWY-5838	Superpathway of menaquinol-8 biosynthesis I	0.0594|96.5	0.0548|100	−0.689 (0.183)	2.49 × 10^−4^	0.017
PWY-5840	Superpathway of menaquinol-7 biosynthesis	0.0506|96.5	0.0472|98.6	−0.737 (0.233)	0.002	0.037
PWY-5845	Superpathway of menaquinol-9 biosynthesis	0.0439|96.5	0.0406|98.6	−0.639 (0.194)	1.25 × 10^−3^	0.028
PWY-5861	Superpathway of demethylmenaquinol-8 biosynthesis I	0.0413|96.5	0.0378|100	−0.739 (0.196)	2.34 × 10^−4^	0.017
PWY-5862	Superpathway of demethylmenaquinol-9 biosynthesis	0.0309|96.5	0.028|98.6	−0.679 (0.204)	1.09 × 10^−3^	0.028
PWY-5897	Superpathway of menaquinol-11 biosynthesis	0.0594|96.5	0.0548|100	−0.704 (0.193)	3.62 × 10^−4^	0.017
PWY-5898	Superpathway of menaquinol-12 biosynthesis	0.0594|96.5	0.0548|100	−0.704 (0.193)	3.62 × 10^−4^	0.017
PWY-5899	Superpathway of menaquinol-13 biosynthesis	0.0594|96.5	0.0548|100	−0.704 (0.193)	3.62 × 10^−4^	0.017
PWY-5913	Partial TCA cycle (obligate autotrophs)	0.0726|100	0.0604|100	−0.639 (0.212)	0.003	0.050
PWY-5918	Superpathway of heme b biosynthesis from glutamate	0.0248|98.8	0.027|97.1	−0.677 (0.234)	0.004	0.070
PWY-6285	Superpathway of fatty acid biosynthesis (*E. coli*)	0.0615|89.5	0.0738|95.7	−0.691 (0.211)	1.32 × 10^−3^	0.028
PWY-6531	Mannitol cycle	0.0194|97.7	0.0197|97.1	−1.014 (0.3)	9.40 × 10^−4^	0.028
PWY66-389	Phytol degradation	0.0071|87.2	0.0073|91.4	−1.825 (0.559)	1.36 × 10^−3^	0.028
PWY-6961	L-ascorbate degradation II (bacterial, aerobic)	0.0137|96.5	0.0169|94.3	−0.937 (0.310)	0.003	0.050
PWY-7385	1,3-propanediol biosynthesis (engineered)	0.0095|77.9	0.0101|74.3	−1.601 (0.561)	0.005	0.076

* A LinDA-linear mixed-effect model was conducted for the relative abundance of microbial metabolic pathways with adjustment for age, gender, BMI, and intake of poultry and legumes. We limited our analysis to 366 metabolic pathways (515) with a prevalence > 10% and a median of relative abundance > 0.001%. Abbreviation: RA: relative abundance; FDR: false discovery rates; Pre: prevalence.

**Table 6:** Association of peanut intervention with the stability of individual microbial taxa and microbial metabolic pathways ^a^.

	Median of Stability				
	Control (n = 43)	Intervention (n = 35)	β (SE)	*p*	FDR
Microbial taxa					
Phylum *Bacteroidota*					
Species MGYG-HGUT-00855	0.763	0.882	0.954 (0.323)	0.003	0.084
Species MGYG-HGUT-04491	0.625	0.786	0.961 (0.307)	0.002	0.062
Species *Alistipes putredinis*	0.768	0.715	−1.065 (0.299)	3.67 × 10^−4^	0.029
Phylum *Firmicutes* A					
Species MGYG-HGUT-04581	0.814	0.840	1.145 (0.306)	1.81 × 10^−4^	0.029
Species MGYG-HGUT-02992	0.721	0.821	0.883 (0.265)	8.49 × 10^−4^	0.048
Species *Oscillibacter* sp900066435	0.637	0.724	0.986 (0.273)	2.99 × 10^−3^	0.029
Species *Faecalibacterium prausnitzii* F	0.752	0.845	0.901 (0.274)	1.02 × 10^−3^	0.048
Species *Faecalibacterium prausnitzii* H	0.793	0.821	0.686 (0.226)	0.002	0.070
Species MGYG-HGUT-00512	0.695	0.831	0.856 (0.264)	1.18 × 10^−3^	0.048
Species MGYG-HGUT-02809	0.750	0.823	0.933 (0.285)	1.05 × 10^−3^	0.048
Species MGYG-HGUT-03166	0.736	0.855	0.986 (0.264)	1.83 × 10^−4^	0.029
Species MGYG-HGUT-03291	0.778	0.852	0.795 (0.257)	0.002	0.063
Metabolic pathways					
PWY-2942: L-lysine biosynthesis III	0.968	0.936	−0.563 (0.172)	1.03 × 10^−3^	0.078
PWY-5675: nitrate reduction V (assimilatory)	0.587	0.399	1.040 (0.306)	6.73 × 10^−4^	0.068
PWY-6595: superpathway of guanosine nucleotides degradation (plants)	0.767	0.645	−0.982 (0.287)	6.27 × 10^−4^	0.068
PWY-6607: guanosine nucleotides degradation I	0.759	0.643	−0.976 (0.287)	6.77 × 10^−4^	0.068

^a^ Beta regression analysis was performed with adjustments for age, gender, BMI, and intake of poultry and legumes. We limited our analysis to 11 phyla, 11 classes, 24 orders, 42 families, 122 gena, 321 species, and 300 metabolic pathways with a prevalence > 20% and a median of relative abundance > 0.01%. FDR was calculated at each taxonomic level. P_FDR_ < 0.1 is considered statistically significant.

## Data Availability

Data are available on request. The data underlying this article will be shared upon reasonable request to the corresponding author due to privacy or ethical restrictions.

## Supplementary Materials

The following supporting information can be downloaded at https://www.mdpi.com/article/10.3390/nu16193313/s1, Figure S1: Model fitness estimation of the number of species-level sub-communities using Latent Dirichlet Allocation; Figure S2: Boxplots of relative abundance of selected phyla by stool collection time of sample and study group; Table S1: Demographic characteristics and self-reported health issues at enrollment; Table S2: Average daily intake of food groups; Table S3: Change in relative abundance between weeks 16 follow-up and baseline in selected individual microbial taxa; Table S4: Association of peanut intervention with 45 species levels subgroups; Table S5: Change in relative abundance between weeks 16 follow-up and baseline in selected individual microbial metabolic pathways.

## Abbreviations

24-HDRs	24 h dietary recalls
BMI	Body mass index
Clr	Centered log-ratio
DNA	Deoxyribonucleic acid
FDR	False discovery rate
FOBT	Fecal occult blood test
ITMCTR	International Traditional Medicine Clinical Trial Registry
LDA	Latent Dirichlet Allocation
LMM	Linear mixed-effects models
PERMANOVA	Permutational Multivariate Analysis of Variance
REDCap	Research Electronic Data Capture
SCFAs	Short-chain fatty acids
SD	Standard Deviation
SE	Standard Error
VinCAPR	Vietnam Colorectal Cancer and Polyps Research
VUMC	Vanderbilt University Medical Center
UHGG	Unified Human Gastrointestinal Genome
