# Supplementary material for: Influence of Peanut Consumption on the Gut Microbiome: A Randomized Clinical Trial
Source: Nutrients. 2024 Sep 30;16(19):3313. doi: 10.3390/nu16193313 (PMC11478729; doi:10.3390/nu16193313)
Supplement: Supplementary file 1 [file nutrients-16-03313-s001.zip › nutrients-3165397-supplementary.pdf]

Supplementary Table S1: Demographic characteristics and self-reported health issues at enrollment

	Control	Peanut intervention	
	(N=43)	(N=35)	P
	n (%)/Mean ± sd)	n (%)/Mean ± sd)	
Age (years old)	56.2±9.0	54.9±8.6	
Under 50 years old	14 (32.6)	10 (28.6)	0.171
50-59 years old	8 (18.6)	13 (37.1)	
60+ years old	21 (48.8)	12(34.3)	
Gender			
Male	21 (48.8)	21 (60.0)	0.325
Female	22 (51.2)	14 (40.0)	
BMI (kg/m^2)	23.9±2.4	24.3±2.5	
Normal weight (18.5 – 22.9)	16 (37.2)	12 (34.3)	0.427
Asian Overweight (23-24.9)	13 (30.2)	7 (20.0)	
Asian obese (25+)	14 (32.6)	16 (45.7)	
**Self-report during past 3 months**			
Diabetes	4 (9.3)	2 (5.7)	0.686
Hypertension	11 (25.6)	4 (11.4)	0.153
Hyperlipidemia	13 (30.2)	10 (28.6)	0.873
Infection requiring antibiotic treatment	1 (2.3)	2 (5.7)	0.585
Health issue changed diet habits	2 (4.7)	0 (0.0)	0.499
Health issue changed bowel movements	4 (9.3)	0 (0.0)	0.123
Food allergy	1 (2.3)	2 (5.7)	0.585
Constipation	2 (4.7)	0 (0.0)	0.499
Gain or loss of more than 2.5 kg of weight	0 (0.0)	0 (0.0)	1.000

Supplementary Table S2: Average daily intake of food groups

Food groups	Control		P value[1]	Intervention		P value[1]	Control	Intervention	P value[2]
	Baseline	Weeks 16 Follow-up		Baseline	Weeks 16 Follow-up		Combined baseline & weeks 16 FU	Combined baseline & weeks 16 FU	
	N=43	N=43		N=35	N=35		N=43	N=35	
	Mean ± sd	Mean ± sd		Mean ± sd	Mean ± sd		Mean ± sd	Mean ± sd	
Grain (g/d)	299.0±91.7	283.1±94.5	0.279	295.4±94.2	286.2±105.9	0.461	291.1±80.1	209.8±93.4	0.989
Staple foods (g/d)	14.6±28.8	10.1±22.9	0.277	8.1±18.8	6.2±19.2	0.549	12.3±18.2	7.1±13.3	0.102
Fruit (g/d)	149.1±112.1	125.1±105.5	0.234	101.4±89.0	123.4±100.5	0.403	137.1±93.3	112.4±73.5	0.285
Vegetables (g/d)	96.7±66.6	111.5±84.8	0.391	96.2±73.2	75.2±65.5	0.065	104.1±53.6	85.7±52.9	0.069
Legumes (g/d)	7.2±19.0	7.2±21.1	0.613	5.2±15.0	4.1±11.7	0.715	7.5±14.2	4.6±8.9	0.661
Red meats (g/d)	78.0±51.6	87.6±66.5	0.491	90.6±94.9	78.5±57.7	0.812	82.8±44.2	84.6±65.3	0.648
Poultry (g/d)	37.7±51.5	72.1±74.0	0.023	53.8±60.4	57.3±65.9	0.773	54.9±47.5	55.5±47.9	0.844
Fish & Shrimp (g/d)	54.5±53.9	40.1±35.9	0.095	63.4±86.6	55.0±58.0	0.870	47.3±39.0	59.2±48.1	0.280
Eggs (g/d)	17.4±19.4	14.5±17.5	0.458	12.3±17.2	18.5±26.6	0.347	16.0±14.0	15.4±16.9	0.596
Dairy products (g/d)	25.6±58.5	18.0±49.5	0.220	28.6±47.2	23.7±64.5	0.251	21.8±47.9	26.2±47.2	0.686
Soybean products (g/d)	18.4±37.8	11.8±21.1	0.270	7.1±16.4	8.0±16.0	0.656	15.1±21.3	7.5±10.6	0.081
Another nut* (g/d)	0.4±2.5	0.5±2.6	0.573	0.3±1.7	0.0±0.0	0.317	0.4±1.8	0.1±0.8	0.408

[1] *Comparing the mean of average daily nutrient intake between 24HDR at baseline and 24HDR at the 16-week follow-up: Paired T-tests were used for food groups with normal distribution; Wilcoxon signed-rank tests were used for food groups with no normal distribution.*

[2] *Comparing the mean of average daily nutrient intake combined 24HDR at baseline and post-16 weeks between control and intervention: T-tests were used for food groups with normal distribution; Wilcoxon rank-sum tests were used for food groups with no normal distribution.*

** Other nuts, including walnuts, lotus seeds, pumpkin seeds, etc. Legume: beans and Peas*

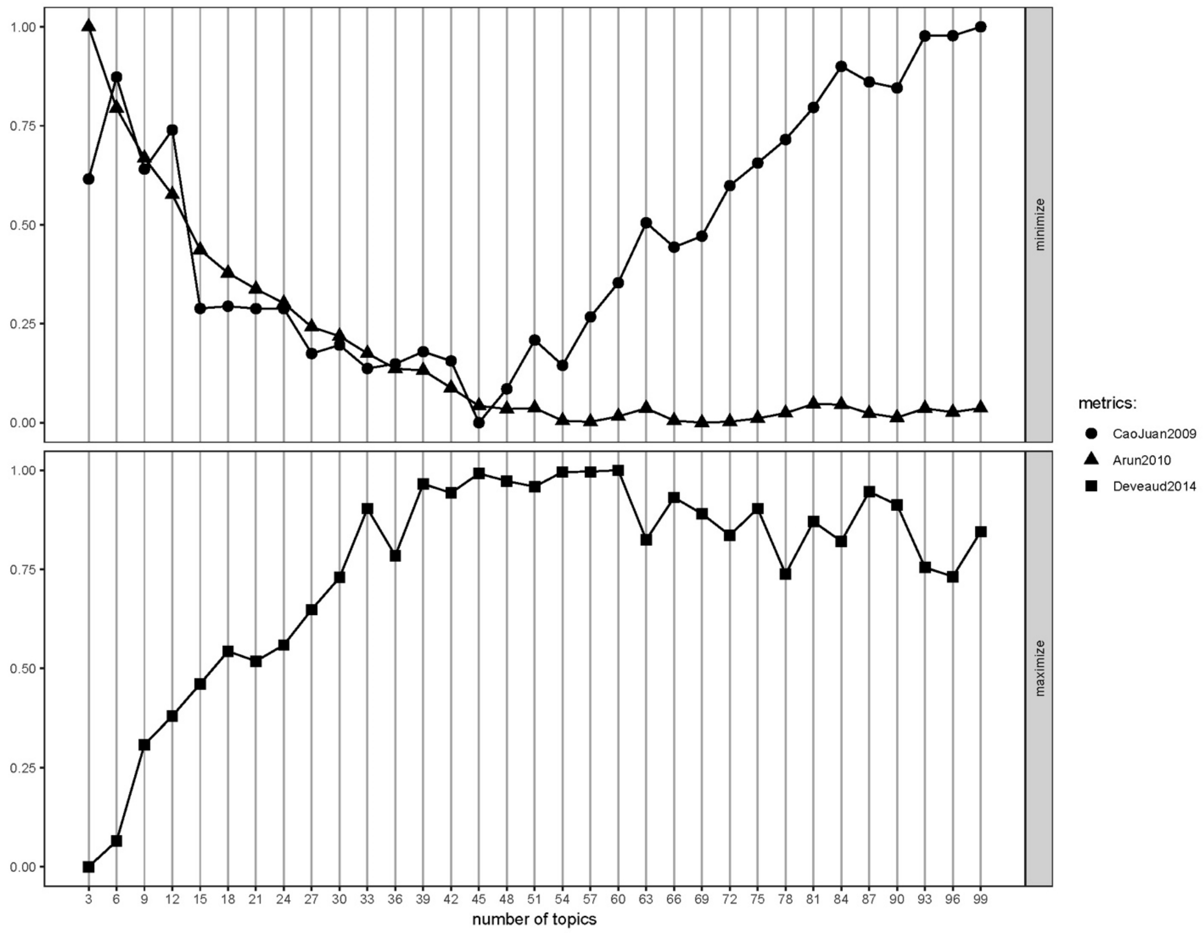

Supplementary Figure S1: Model fitness estimation of the number of species-level sub-communities using Latent Dirichlet Allocation

Supplementary Table S3. Change in relative abundance between weeks 16 follow-up and baseline in selected individual microbial taxa

	Control		Intervention		Change between 16w-FU and baseline	
	Baseline	16w-FU	Baseline	16w-FU	Control	Intervention
**Microbial taxa**	RA, median (%)	RA, median (%)	RA, median (%)	RA, median (%)	Absolute change, median (%)	Absolute change, median (%)
Phylum *Actinobacteriota*	2.6	3.0	2.4	2.1	0.2	-0.1
Class *Coriobacteriia*	1.9	2.1	1.8	1.5	0.2	-0.3
Order *Coriobacteriales*	1.9	2.1	1.8	1.5	0.2	-0.3
Phylum *Firmicutes*	2.9	3.5	2.8	2.8	0.5	0.2
Class *Bacilli*	2.9	3.5	2.8	2.8	0.5	0.2
Order *Erysipelotrichales*	1.7	1.7	1.4	1.8	0.0	0.2
Phylum *Firmicutes A*	53.0	55.4	50.6	48.9	2.4	-1.6
Class *Clostridia*	53.0	55.4	50.6	48.9	2.4	-1.6
Phylum *Proteobacteria*	1.4	1.2	1.4	1.2	0.2	0.0
Class *Gammaproteobacteria*	1.4	1.2	1.4	1.2	0.2	0.0
Phylum *Synergistota*	1.25×10^{-3}	1.15×10^{-3}	0.0	0.0	0.0	0.0
Class *Synergistia*	1.25×10^{-3}	1.15×10^{-3}	0.0	0.0	0.0	0.0

RA: Relative abundance

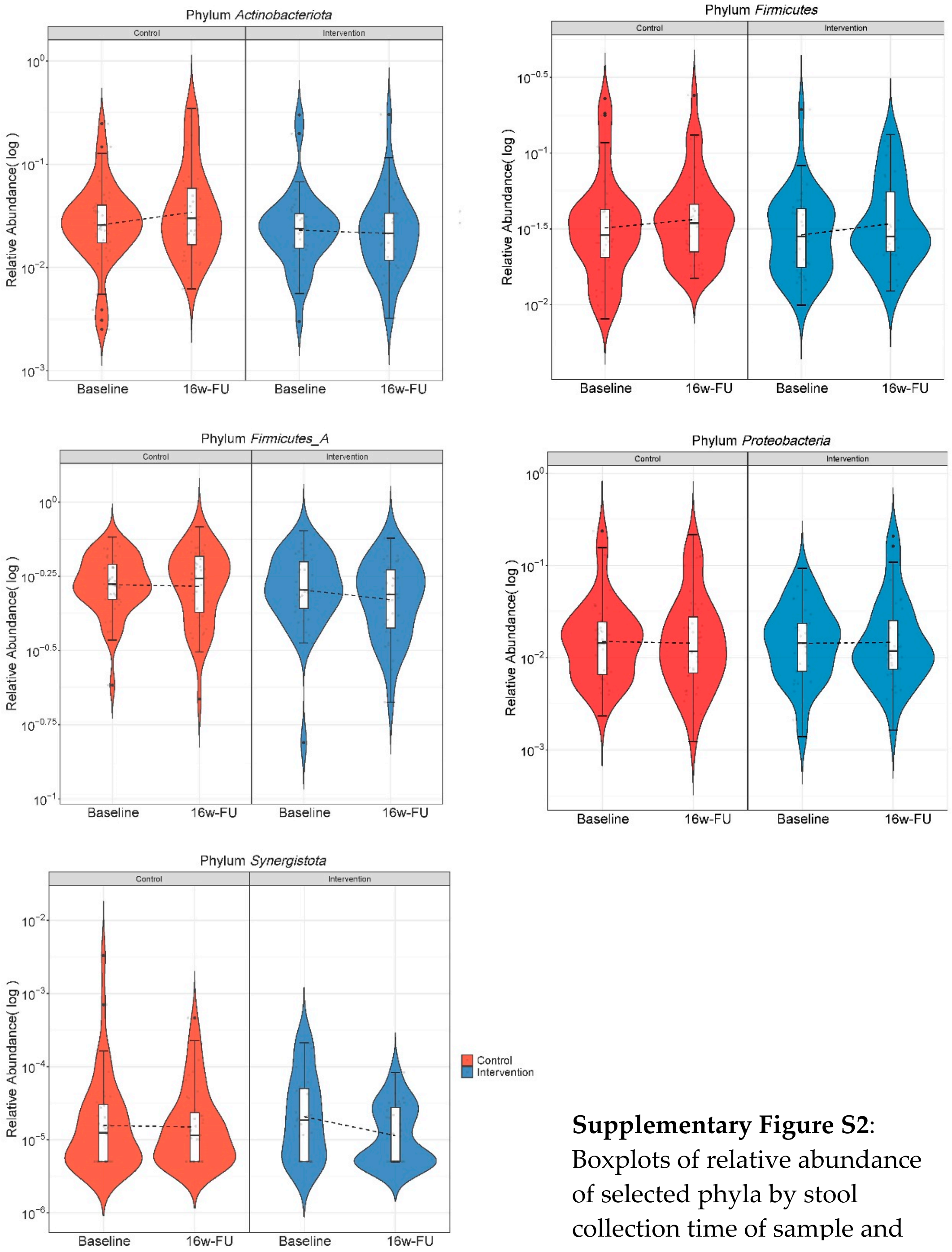

Supplementary Figure S2: Boxplots of relative abundance of selected phyla by stool collection time of sample and

Supplementary Table S4: Association of peanut intervention with 45 species levels subgroups

Subgroup and top 5 species	*β (SE)*	*P*	*FDR*
**Subgroup 1**	-4.633 (1.415)	1.33×10^{-3}	0.020
MGYG-HGUT-00200 (53.34%), Faecalicatena faecis (11.75%), Anaerostipes hadrus (2.94%), Blautia A sp900066165 (2.56%), MGYG-HGUT-02772 (1.62%)			
**Subgroup 2**	-1.824 (1.223)	0.138	0.398
Faecalibacterium prausnitzii G (38.92%), Faecalicatena faecis (19.10%), Bacteroides uniformis (3.99%), Bacteroides A plebeius (2.96%), Parabacteroides distasonis (2.89%)			
**Subgroup 3**	-2.329 (1.836)	0.207	0.489
CAG-127 sp900319515 (24.86%), Ruminococcus C callidus (8.00%), Lachnospira rogosae (6.99%), Acetatifactor sp900066365 (4.34%), Blautia A wexlerae (4.15%)			
**Subgroup 4**	-0.082 (1.545)	0.958	0.991
Prevotella copri A (31.49%), MGYG-HGUT-02603 (10.89%), MGYG-HGUT-03347 (9.28%), MGYG-HGUT-02834 (4.48%), MGYG-HGUT-00644 (3.77%)			
**Subgroup 5**	0.057 (1.672)	0.973	0.991
Bacteroides eggerthii (14.19%), Bacteroides uniformis (12.47%), Bacteroides B dorei (6.97%), Blautia A wexlerae (5.37%), Bacteroides B massiliensis (4.08%)			
**Subgroup 6**	-4.629 (1.962)	0.020	0.110
Megamonas funiformis (32.72%), Prevotella copri (14.52%), Prevotella sp000436915 (4.72%), Faecalibacterium prausnitzii C (3.32%), Blautia A massiliensis (2.63%)			
**Subgroup 7**	-6.125 (1.804)	8.85×10^{-4}	0.020
Roseburia inulinivorans (24.12%), Bacteroides B dorei (4.33%), Faecalicatena torques (3.93%), Holdemanella sp002299315 (3.71%), Dorea longicatena B (3.34%)			
**Subgroup 8**	0.9 (1.589)	0.572	0.786
Faecalibacterium prausnitzii J (14.12%), Prevotella copri (9.63%), Bacteroides B dorei (8.52%), MGYG-HGUT-03699 (3.75%), KLE1615 sp900066985 (3.43%)			
**Subgroup 9**	-1.359 (2.184)	0.535	0.786
Bacteroides B dorei (30.17%), Bacteroides stercoris (21.64%), Megamonas funiformis (15.55%), Faecalicatena gnavus (4.91%), Parabacteroides distasonis (4.41%)			
**Subgroup 10**	-0.939 (1.793)	0.601	0.793
Eubacterium R sp000436835 (19.31%), Agathobacter rectale (9.93%), Coprococcus B comes (5.11%), Dorea longicatena (3.86%), Faecalibacterium prausnitzii K (3.69%)			
**Subgroup 11**	-3.111 (1.691)	0.068	0.304

Subgroup and top 5 species	*β (SE)*	*P*	*FDR*
Blautia A wexlerae (20.95%), Fusicatenibacter saccharivorans (16.09%), Anaerostipes hadrus (5.05%), CAG-56 sp900066615 (3.31%), Eubacterium E hallii A (2.96%)			
**Subgroup 12**	-2.562 (1.489)	0.087	0.304
Streptococcus salivarius (32.07%), Streptococcus vestibularis (11.10%), Streptococcus sp001556435 (5.90%), CAG-56 sp900066615 (4.92%), Dorea longicatena B (4.75%)			
**Subgroup 13**	0.71 (1.417)	0.617	0.793
Prevotella copri A (7.95%), Prevotella stercorea (6.36%), Blautia A wexlerae (4.17%), Ruminococcus D bicirculans (3.95%), Acetatifactor sp900066365 (3.44%)			
**Subgroup 14**	0.021 (1.922)	0.991	0.991
Faecalibacterium prausnitzii E (13.58%), Blautia sp000436935 (9.38%), Lachnospira eligens B (4.87%), KLE1615 sp900066985 (4.15%), TF01-11 sp003529475 (3.48%)			
**Subgroup 15**	-2.838 (1.085)	9.84×10^{-3}	0.074
Bifidobacterium pseudocatenulatum (66.15%), Anaerostipes hadrus (6.14%), Fusicatenibacter saccharivorans (3.62%), Dorea longicatena B (3.12%), Faecalibacterium prausnitzii D (1.80%)			
**Subgroup 16**	-0.313 (1.443)	0.829	0.91
Faecalibacterium prausnitzii C (12.00%), Blautia A wexlerae (6.24%), Blautia A massiliensis (4.26%), Blautia A sp900066165 (4.11%), Fusicatenibacter saccharivorans (3.61%)			
**Subgroup 17**	-1.888 (1.7)	0.269	0.576
Faecalicatena faecis (16.60%), Prevotella copri (8.99%), Faecalibacterium prausnitzii K (5.18%), Faecalicatena lactaris (3.71%), Fusicatenibacter saccharivorans (3.45%)			
**Subgroup 18**	-4.741 (1.98)	0.018	0.110
Bacteroides B massiliensis (34.24%), Bacteroides B dorei (14.05%), Faecalicatena torques (10.41%), Bacteroides B sartorii (3.12%), MGYG-HGUT-02610 (3.10%)			
**Subgroup 19**	-0.864 (1.544)	0.577	0.786
Prevotella copri (41.57%), Megamonas funiformis (10.59%), Prevotella copri A (6.36%), MGYG-HGUT-04456 (3.05%), Alistipes putredinis (2.39%)			
**Subgroup 20**	1.555 (1.777)	0.383	0.690
Lachnospira eligens B (18.31%), Bacteroides B dorei (16.96%), Bacteroides caccae (11.31%), Lachnospira sp003537285 (4.84%), MGYG-HGUT-03899 (2.67%)			
**Subgroup 21**	-4.664 (1.351)	7.23×10^{-4}	0.020
Escherichia coli D (63.50%), Escherichia fergusonii (6.90%), Escherichia sp000208585 (3.76%), Bacteroides stercoris (2.37%), Lachnospira rogosae (1.95%)			
**Subgroup 22**	2.89 (1.714)	0.094	0.304
Prevotella copri (19.60%), Alistipes putredinis (10.07%), Blautia A obeum (2.78%), Blautia A sp900066165 (2.62%), KLE1615 sp900066985 (2.34%)			
**Subgroup 23**	0.904 (1.574)	0.567	0.786

Subgroup and top 5 species	*β (SE)*	*P*	*FDR*
Prevotella sp000434515 (30.04%), Prevotella copri (16.45%), MGYG-HGUT-04456 (3.67%), Acetatifactor sp900066365 (2.65%), MGYG-HGUT-00200 (2.00%)			
**Subgroup 24**	-4.91 (1.733)	5.26×10^{-3}	0.059
Ruminococcus D bicirculans (26.69%), Faecalibacterium prausnitzii D (11.07%), Fusicatenibacter saccharivorans (10.02%), Blautia A sp900066165 (4.28%), Bacteroides stercoris (3.45%)			
**Subgroup 25**	2.192 (1.554)	0.161	0.425
Prevotella sp000436035 (20.77%), Prevotella copri (9.93%), MGYG-HGUT-00644 (7.46%), Prevotella sp002265625 (4.02%), Prevotellamassilia timonensis (3.39%)			
**Subgroup 26**	-2.979 (1.699)	0.082	0.304
Ruminococcus E bromii B (54.20%), Ruminococcus E bromii (14.56%), Bacteroides B dorei (4.49%), Bacteroides ovatus (2.17%), Blautia A obeum (2.13%)			
**Subgroup 27**	0.416 (1.875)	0.825	0.910
Bacteroides uniformis (15.74%), Bacteroides stercoris (13.28%), MGYG-HGUT-00200 (7.66%), Bacteroides B dorei (5.22%), Acetatifactor sp900066565 (4.96%)			
**Subgroup 28**	-1.564 (1.56)	0.318	0.622
Agathobacter rectale (65.63%), MGYG-HGUT-02670 (10.16%), Fusicatenibacter saccharivorans (1.55%), Blautia A massiliensis (1.33%), MGYG-HGUT-01186 (1.05%)			
**Subgroup 29**	0.833 (1.887)	0.66	0.825
Prevotella copri (72.58%), MGYG-HGUT-04456 (3.73%), MGYG-HGUT-00644 (2.22%), Prevotella sp000434515 (1.61%), MGYG-HGUT-00553 (1.55%)			
**Subgroup 30**	1.065 (1.454)	0.465	0.776
Megamonas funiformis (9.87%), Faecalicatena gnavus (7.62%), Blautia A wexlerae (7.49%), Prevotella sp000436915 (6.87%), MGYG-HGUT-02303 (6.17%)			
**Subgroup 31**	-2.28 (1.542)	0.142	0.398
Bacteroides A plebeius A (68.78%), Bacteroides A plebeius (11.73%), Faecalicatena faecis (4.32%), Bacteroides uniformis (2.47%), Anaerostipes hadrus (1.17%)			
**Subgroup 32**	-0.854 (1.036)	0.411	0.712
Prevotella sp000434975 (47.92%), Prevotella stercorea (3.96%), Blautia A wexlerae (3.82%), Faecalibacterium prausnitzii E (3.48%), MGYG-HGUT-03899 (2.08%)			
**Subgroup 33**	-2.137 (1.77)	0.229	0.515
Faecalicatena faecis (13.99%), Romboutsia timonensis (9.64%), MGYG-HGUT-00839 (9.11%), CAG-56 sp900066615 (7.42%), Dorea longicatena B (4.45%)			
**Subgroup 34**	1.031 (1.531)	0.502	0.786

Subgroup and top 5 species	*β (SE)*	*P*	*FDR*
Prevotella sp002265625 (27.80%), Faecalicatena faecis (5.84%), Prevotellamassilia timonensis (5.39%), Prevotella copri A (4.37%), Roseburia inulinivorans (3.55%)			
**Subgroup 35**	-4.001 (1.450)	$6.56x10^{-3}$	0.059
Holdemanella biformis (30.75%), Blautia A wexlerae (8.45%), Holdemanella sp002299315 (3.05%), Dorea formicigenerans (2.76%), Dorea longicatena (2.51%)			
**Subgroup 36**	-3.409 (1.562)	0.031	0.153
Klebsiella pneumoniae (47.50%), Holdemanella sp002299315 (3.64%), MGYG-HGUT-03899 (2.78%), MGYG-HGUT-00245 (2.37%), Bacteroides B dorei (2.31%)			
**Subgroup 37**	0.584 (1.593)	0.715	0.869
Acetatifactor sp900066565 (21.38%), Bacteroides B massiliensis (10.99%), Faecalibacterium sp003449675 (6.03%), MGYG-HGUT-00245 (4.91%), Species KLE1615 sp900066985 (3.88%)			
**Subgroup 38**	-0.602 (1.827)	0.742	0.879
Bacteroides B sartorii (23.86%), Bacteroides thetaiotaomicron (13.79%), Bacteroides uniformis (8.12%), Parabacteroides merdae (7.99%), Faecalicatena gnavus (7.26%)			
**Subgroup 39**	-2.002 (2.114)	0.345	0.647
Faecalicatena gnavus (40.99%), Tyzzerella nexilis (10.94%), Blautia hansenii (5.63%), Dorea longicatena B (4.36%), Blautia A sp900066165 (2.85%)			
**Subgroup 40**	0.046 (1.553)	0.976	0.991
Agathobacter faecis (38.45%), Roseburia intestinalis (6.52%), MGYG-HGUT-03899 (6.06%), Fusicatenibacter saccharivorans (5.78%), Faecalibacterium prausnitzii D (5.33%)			
**Subgroup 41**	0.321 (1.471)	0.828	0.910
MGYG-HGUT-00644 (17.66%), Prevotella sp900313215 (6.97%), MGYG-HGUT-04456 (5.48%), Prevotella copri (4.05%), Acetatifactor sp900066565 (3.29%)			
**Subgroup 42**	-2.391 (1.785)	0.183	0.456
Bacteroides B dorei (47.01%), Blautia A wexlerae (4.45%), Bacteroides stercoris (4.42%), Bacteroides thetaiotaomicron (4.05%), Parabacteroides distasonis (4.03%)			
**Subgroup 43**	-3.14 (1.866)	0.095	0.304
Bacteroides A coprocola (31.36%), MGYG-HGUT-02275 (7.77%), Bacteroides B dorei (7.33%), Bacteroides B massiliensis 6.94%), Fusicatenibacter saccharivorans (4.77%)			
**Subgroup 44**	-2.042 (2.003)	0.31	0.622
Megamonas funiformis (17.64%), Bacteroides A plebeius (15.08%), Roseburia intestinalis (7.78%), Lachnospira rogosae (6.37%), Roseburia inulinivorans (6.29%)			
**Subgroup 45**	-1.076 (1.68)	0.523	0.786

Subgroup and top 5 species	*β (SE)*	*P*	*FDR*
Coprococcus sp900066115 (21.08%), Ruminococcus C callidus (5.52%), Fusicatenibacter saccharivorans (3.10%), Dorea longicatena B (2.88%), Holdemanella_sp002299315 (2.74%)			

*LinDA Linear Mixed-effect Models were conducted with adjustment for age, gender, BMI, and intake of poultry and legumes.

Supplementary Table S5. Change in relative abundance between weeks 16 follow-up and baseline in selected individual microbial metabolic pathways

		Control		Intervention		Change between 16w-FU and baseline	
		Baseline	16w-FU	Baseline	16w-FU	Control	Intervention
	**Microbial metabolic pathways**	RA, median (%)	RA, median (%)	RA, median (%)	RA, median (%)	Absolute change, median (%)	Absolute change, median (%)
NAD-BIOSYNTHESIS-II	NAD salvage pathway III (to nicotinamide riboside)	0.014	0.013	0.011	0.010	0.002	0.003
P461-PWY	Hexitol fermentation to lactate, formate, ethanol and acetate	0.083	0.093	0.075	0.083	0.023	0.006
P4-PWY	Superpathway of L-lysine, L-threonine and L-methionine biosynthesis I	0.060	0.062	0.055	0.059	0.003	0.008
PWY0-301	L-ascorbate degradation I (bacterial, anaerobic)	0.015	0.016	0.021	0.019	0.001	0.002
PWY0-781	Aspartate superpathway	0.064	0.065	0.058	0.062	0.004	0.008
PWY-5675	Nitrate reduction V (assimilatory)	0.011	0.011	0.016	0.016	0.002	0.006
PWY-5705	Allantoin degradation to glyoxylate III	0.002	0.001	0.002	0.002	0.000	0.000
PWY-5723	Rubisco shunt	0.026	0.021	0.026	0.026	-0.001	0.004
PWY-5837	2-carboxy-1,4-naphthoquinol biosynthesis	0.021	0.025	0.023	0.018	0.004	0.004
PWY-5838	Superpathway of menaquinol-8 biosynthesis I	0.059	0.060	0.066	0.048	0.001	-0.001
PWY-5840	Superpathway of menaquinol-7 biosynthesis	0.046	0.054	0.047	0.047	0.008	8.82×10^{-5}
PWY-5845	Superpathway of menaquinol-9 biosynthesis	0.044	0.043	0.052	0.040	-0.001	0.003
PWY-5861	Superpathway of demethylmenaquinol-8 biosynthesis I	0.041	0.041	0.046	0.033	0.001	-0.001
PWY-5862	Superpathway of demethylmenaquinol-9 biosynthesis	0.031	0.030	0.036	0.028	-0.001	0.002
PWY-5897	Superpathway of menaquinol-11 biosynthesis	0.054	0.062	0.061	0.046	0.002	-0.001
PWY-5898	Superpathway of menaquinol-12 biosynthesis	0.054	0.062	0.061	0.046	0.002	-0.001
PWY-5899	Superpathway of menaquinol-13 biosynthesis	0.054	0.062	0.061	0.046	0.002	-0.001
PWY-5913	Partial TCA cycle (obligate autotrophs)	0.066	0.090	0.061	0.060	0.018	0.003

PWY-5918	Superpathway of heme b biosynthesis from glutamate	0.025	0.024	0.031	0.027	0.001	-0.001
PWY-6285	Superpathway of fatty acids biosynthesis (E. coli)	0.064	0.056	0.072	0.076	$2.42x10^{-4}$	0.006
PWY-6531	Mannitol cycle	0.018	0.020	0.015	0.020	0.001	0.001
PWY66-389	Phytol degradation	0.005	0.009	0.007	0.007	0.004	-0.001
PWY-6961	L-ascorbate degradation II (bacterial, aerobic)	0.014	0.013	0.016	0.017	0.001	0.003
PWY-7385	1,3-propanediol biosynthesis (engineered)	0.011	0.005	0.007	0.014	0.000	0.007

RA: Relative abundance
